# Musical emotions affect memory for emotional pictures

**DOI:** 10.1038/s41598-022-15032-w

**Published:** 2022-06-23

**Authors:** Francesca Talamini, Greta Eller, Julia Vigl, Marcel Zentner

**Affiliations:** Department of Psychology, University of Innsbruck, Innsbruck, Austria

**Keywords:** Psychology, Human behaviour

## Abstract

Music is widely known for its ability to evoke emotions. However, assessing specific music-evoked emotions other than through verbal self-reports has proven difficult. In the present study, we explored whether mood-congruency effects could be used as indirect measures of specific music-evoked emotions. First, participants listened to 15 music excerpts chosen to induce different emotions; after each excerpt, they were required to look at four different pictures. The pictures could either: (1) convey an emotion congruent with that conveyed by the music (i.e., congruent pictures); (2) convey a different emotion than that of the music, or convey no emotion (i.e., incongruent pictures). Second, participants completed a recognition task that included *new* pictures as well as *already seen* congruent and incongruent pictures. From previous findings about mood-congruency effects, we hypothesized that if music evokes a given emotion, this would facilitate memorization of pictures that convey the same emotion. Results revealed that accuracy in the recognition task was indeed higher for emotionally congruent pictures than for emotionally incongruent ones. The results suggest that music-evoked emotions have an influence on subsequent cognitive processing of emotional stimuli, suggesting a role of mood-congruency based recall tasks as non-verbal methods for the identification of *specific* music-evoked emotions.

## Introduction

Music-evoked emotions have grown into a key area of research in the psychology of music^1^, and they have also gained a significant place in the psychology of emotions and in affective neuroscience. Its growing role across these fields has compelled researchers to critically examine measurement approaches to music-induced emotions^2–5^. Among the most frequently used approaches are subjective verbal reports of emotions on the one hand (e.g.,^6^) and physiological measures on the other (e.g.,^3^).

Measuring music-induced emotions by use of verbal reports has the advantage of capturing specific, musically relevant emotional states, such as awe, tenderness, nostalgia, or sadness. The disadvantage is that the extent to which these emotions are actually felt remains often unclear. When reporting a given emotion, listeners may describe the stereotypical emotional connotation of the music that they perceived, rather than an emotion that they have truly felt^5,6^. While perceived and felt emotions are often correlated^7^, they do not necessarily have to coincide: an individual who listens to sad music can feel pleasure and positive emotions even if the music has negative valence^8^.

Physiological measures, such as skin conductance or heart rate, have a greater probability of capturing emotional arousal (i.e., felt emotions), but they lack the specificity that would be necessary to identify specific emotional states, such as awe, nostalgia, or sadness. There is evidence suggesting that neuroimaging methods may provide more specific emotional information than do psychophysiological measures^9^. However, such methods are time- and resource-intensive, and may therefore be difficult to use on a broad scale. A non-physiological method capable of identifying specific induced emotions, would allow broad, cost-effective assessment of emotional experiences with music. This step would broaden the basis for measuring music-induced emotion, including via online experiments that do not easily accommodate the use of physiological measures. Moreover, such a method would be comparatively easy and quick to analyze with respect to physiological assessment methods. In this study, we aimed to study music-evoked emotions behaviorally, by using a paradigm that leverages mood-congruency effects on attention and memory.

The affective state of an individual can influence the perception of external stimuli, where to direct attention, and what will be remembered, and these phenomena are commonly referred to as “mood congruency effects”^10–12^. Previous research has shown that information processing is facilitated when the affective tone of the information matches the perceiver’s mood. For example, a study by Niedenthal and Setterlund^13^ showed that the affective state of the participants facilitated the recognition of emotionally-congruent words. Furthermore, Becker and Leinenger^10^ observed that participants were more likely to detect the appearance of an unexpected face during a tracking task when the expression of the face matched the participants’ mood. Other findings showed that individuals in a positive affective state, compared to a neutral one, direct attention to peripheral positive stimuli^14^, and more often towards rewarding than to aversive information^15^. Demonstrations of affective state influences on cognitive processes also come from clinical population studies (e.g., in mood disorders)^16–18^. For example, dysphoric patients hold attention for longer to negative stimuli^19^ and they show increased neural response to sad stimuli^20^, suggesting that they attend to information that matches their negative mood.

The semantic associative network model of memory by Bower^21^ provides a possible explanation for these mood-congruency effects. According to the model, memory can be seen as a network of nodes and connections. Each emotion has its specific node. Connected to this node are other nodes containing events, verbal labels, and any kind of information that is associated with that specific emotion. Once the emotional node is activated, the other nodes connected to it will become more easily accessible to attention and/or memory. It is thus expected that when an individual is in a certain affective state, this state will make mood-congruent categories more salient than incongruent ones, thus influencing where to direct attention and/or what to retrieve.

Music has a successful record as a mood-induction procedure (for a review, see^22^) and studies have shown that emotions can arise in the subject after only 8 s of listening^23^. Concerning the use of music to investigate mood-congruency effects, most of evidence comes from studies based on free recall tasks. For example, some studies asked their participants to recall autobiographical memories after having listened to emotional music^24,25^. Analysing autobiographical memories in terms of emotional content is not straightforward, however, and may leave considerable room for interpretation. Although studies investigating the influence of music-induced emotions on memory processes with more objective tasks (e.g., recall of previously presented words) exist, they had several limitations. One is the use of between-subjects designs (i.e., different participants were allocated to different emotion-inducing conditions)^26^, another that only one type of emotion was tested (e.g., sadness)^27^. For example, Tesoriero and Rickard^28^ investigated mood-congruency effects by using music to induce an emotional state in their participants. Specifically, they selected music to induce four different emotions (i.e., calmness, happiness, sadness, and fear) in four different groups of participants. Later, they tested whether the induced emotions affected the recall of emotionally congruent and incongruent narrative content. The authors observed mood-congruency effects in only one condition, that is, a superior recall of positive information over negative information in the group that listened to happy music.

As aforementioned, music can convey emotions and, eventually, also change the affective state of the individual^6,9,29,30^. We can thus hypothesize that if an individual experiences an emotion in response to music, this could indirectly affect his/her cognitive processes, as already observed in the study by Tesoriero and Rickard^28^. Based on this hypothesis, in the present study, all participants listened to some music excerpts (conveying three different types of music-specific emotions), and then looked at different pictures that were either congruent (i.e., convey the same emotion as that conveyed by the music) or incongruent (i.e., convey a different emotion from that of the music, or convey no particular emotion). In a second part, we administered a recognition task with 50% of the pictures previously presented and 50% of new pictures. In the case participants experienced the emotion conveyed by the music, we expected this to facilitate the memorization of emotional congruent pictures, in comparison to incongruent pictures.

We chose to include music excerpts that were categorized based on music-specific emotions. In fact, emotional responses to music may not be fully captured by generic emotion models, such as the *circumplex* model, which posits that all emotions can be represented as points in two-dimensional (arousal by valence) space, or by basic emotion models that were developed for the representation of non-aesthetic, everyday emotions^5^. Indeed, in a series of studies, Zentner et al. found that a music-specific model may capture the richness of emotion induced by music in a more comprehensive and nuanced way^6^. The choice of using pictures for the memory task was made because they can be easily selected to match the emotion evoked by the music (e.g., without needing any interpretation); in fact, they can convey an emotion immediately, other than, for example, using narrative content that would instead require a deeper processing to recognize its emotional connotation.

We also collected information about individual differences that could influence the experience of musical emotions, namely, music expertise, gender, current affective state, and emotional intelligence. Concerning music expertise, previous studies observed that musicians possess superior emotion recognition abilities in music than non-musicians^31^, and that they experience more intense and differentiated musical emotions than non-musicians and amateur musicians^32^. Concerning the current affective state, it is important to consider the mood of participants at the beginning of the experiment, as individuals in a negative mood were found to perceive more sadness in music that did not have a clear negative valence^33,34^. Regarding gender, a meta-analysis showed that females and males differ in terms of recognizing emotions (conveyed by voice, and/or faces/postures), with females showing higher recognition abilities than males^35^. However, less is known about gender differences in the experience of emotions in music, and a few studies did not observe any group difference^21,26^. Finally, we also assessed emotional intelligence as it has shown to be connected to emotion recognition in musical stimuli^36^. Moreover, individuals with higher empathy, a concept that is related to emotional intelligence, are better at perceiving the emotional intentions of musicians, react more intensely to music in general and especially to unfamiliar sad pieces of music than less empathic ones^37–39^.

In summary, we expected that (a) the emotionally congruent pictures with the music would be better remembered than the incongruent ones and that (b) some individual difference variables (e.g., emotional intelligence, music expertise) might increase the accuracy difference between congruent and incongruent pictures.

## Method

### Participants

Two-hundred adults (69 females, 3 non-binary) participated in this study. They were mainly university students, with a mean age of 23.86 years (*SD* = 4.65), recruited via the university mailing list, during university classes, and through posts on social media. Bachelor students of psychology obtained course credits in exchange of their participation. The other participants were not compensated for their participation. One hundred and seventeen participants reported to have some music experience in playing an instrument and/or singing (*M* = 11.08 years, *SD* = 4.66, years range = 3–22), out of which only eight considered themselves “professional musicians”. All participants had a high level of education, with 112 individuals having completed high school, 61 having a bachelor's degree, 26 a master's degree and one person a higher degree.

A post-hoc power analysis (computed with g-power^40^) for a dependent sample t-test, with 200 participants and a medium effect size (i.e., *d* = 0.38, see results section), revealed that we achieved a statistical power of 99%.

### Materials

#### Music

The music excerpts we selected had already been used in previous works on music-evoked emotions and categorized with the Geneva Emotional Music Scale (GEMS)^6,9,41^. The GEMS is a domain-specific scale specifically designed to capture music-evoked emotions. It consists of nine second-order categories (Wonder, Transcendence, Nostalgia, Tenderness, Peacefulness, Joyful Activation, Power, Tension and Sadness) and three first-order emotion factors (Sublimity, Vitality, and Unease). We used the GEMS scale as it is based on categories with musical relevance (e.g., tension, nostalgia) rather than on domain-general, basic emotions categories (e.g., fear, disgust) or on domain-general dimensions (i.e., valence and arousal). Having categories of emotions instead of dimensions seems also important as the affective state of participants was found to facilitate the recognition of emotional congruent stimuli (i.e., words) when they both (i.e., the affective state and the word) belonged to the same emotional category, but not when they had the same valence^13^. We selected 15 music excerpts, equally dived into three categories: two first order categories of the GEMS (i.e., vitality and sublimity), and one second-order category (i.e., tension).

The first-order category “unease” (that together with “tension” includes also “sadness”) was represented by tension only, rather than both tension and sadness, because the intercorrelations between the two have been found to be relatively low^6^. Moreover, the decision of not including a further category for “sadness” was made because sadness in music is often related with some of the emotions of the sublimity category (e.g., nostalgia^26,42^), thus possibly having a less negative connotation than that of a picture conveying sadness. Finally, we decided to use the higher-order categories sublimity, vitality, and unease (represented by tension here) rather than the nine more specific emotional categories for two reasons: first, the narrower the GEMS emotions the more they could be specific to music (e.g., “transcendence” could be hardly elicited by a picture). Secondly, it would have been near impossible to achieve an experimentally viable balance of items/trials, symmetry of picture presentation, and duration of the sessions using nine categories.

The intensities of the files were matched for average RMS amplitude with the software “Cool Edit Pro”. The complete list of the excerpts used is reported in the supplemental material.

#### Pictures

In order for music and pictures to be matched in terms of emotion, the pictures were taken from the EmoMadrid^43^ database (after having been granted permission to use them by the authors) and were pre-tested in a pilot study, in which we asked 98 participants (divided into four groups, each to evaluate 50 pictures) to categorize each picture according to the GEMS-9 scale (with an additional “neutral” option). Because the distinction between feeling an emotion and recognizing was not straightforward for all individuals in the case of pictures, we asked them to select either the emotion that they felt while looking at the picture, or, if they could not feel anything, the emotion they thought the picture was conveying. We then selected 80 pictures that were equally distributed to four categories (i.e., tension, sublimity, vitality, and neutral). The inclusion of the neutral category was needed to have a symmetrical picture presentation on the screen. In fact, with three pictures only, one would have always been more salient that the others.

#### Memory experiment

**Figure 1:**
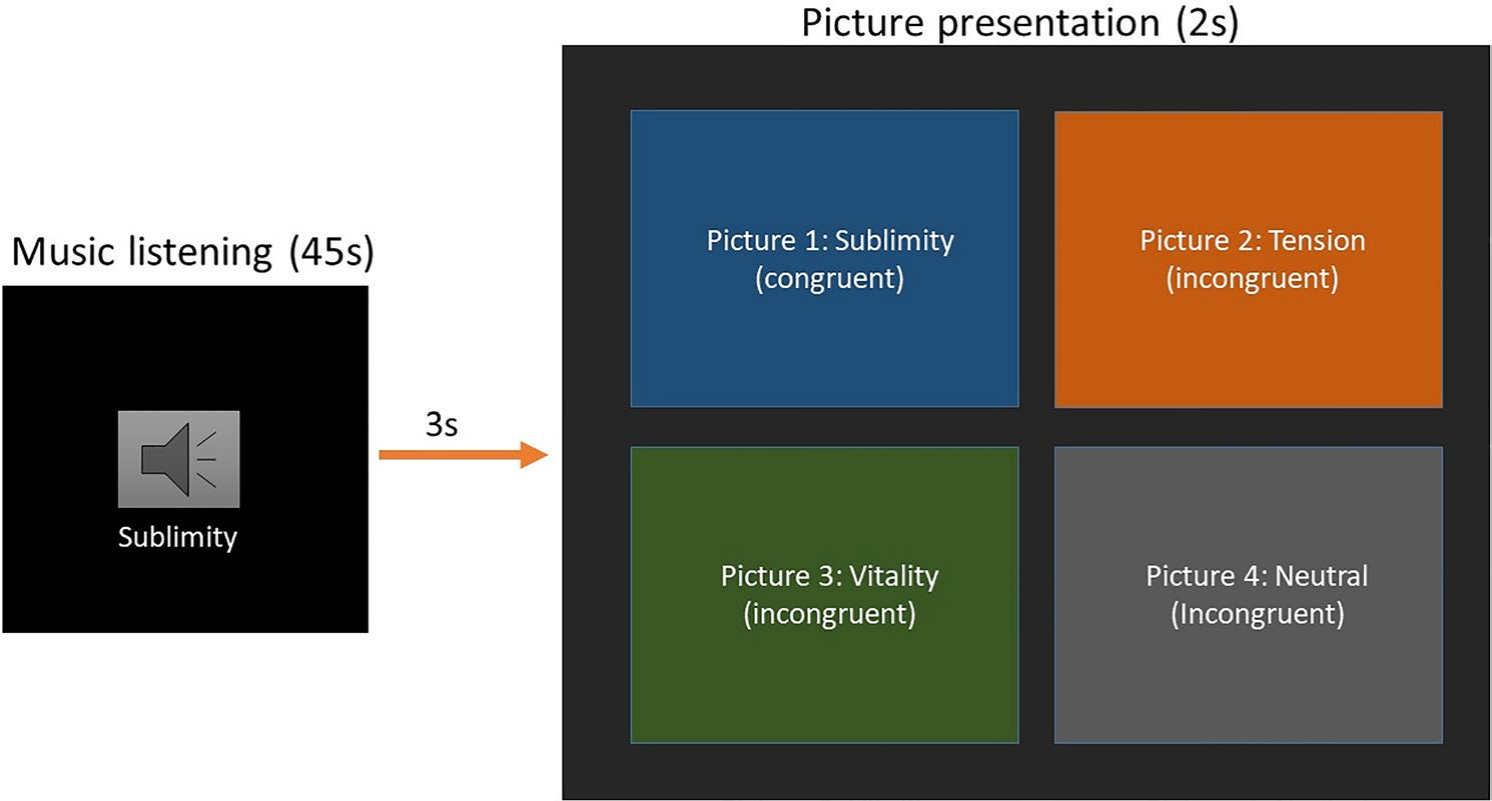
Example of a trial where the participant listens to a musical excerpt belonging to the “sublimity” emotional category, and then looks at the pictures appearing on the screen. Examples of the original pictures cannot be shown because of redistribution limitations imposed by the EmoMadrid database.

The memory experiment was designed specifically for the present study with the program Psychopy^44^. The first part consisted of the presentation of the music and the pictures, and had a total of 15 trials. The 15 trials were equally distributed into three separate blocks, corresponding to the three categories of the GEMS (i.e., tension, sublimity, vitality). Each trial consisted of listening to a Western Classical music excerpt for 45 s, and then looking at four pictures appearing simultaneously on the screen for 2 s. One picture was always congruent with the emotion expressed by the music (i.e., it belonged to the same GEMS category), and three pictures, here defined as “incongruent”, were belonging to the three remaining categories (i.e., the two remaining GEMS categories, and the neutral category). The order of the blocks was randomized across participants as well as the order of the excerpts within each block. The pictures presented as congruent and incongruent were also randomized across participants (e.g., picture 1 could be congruent for one subject, and incongruent for another subject). An example of a trial is depicted in Fig. 1.

After the first part finished, and before the second part began, participants had to solve 4 arithmetic equations, and answer questions about the familiarity with the music and how much they liked it. This part served as a distractor to avoid recency effects on the following memory task. In the second part of the experiment, participants had to perform the recognition task. This task consisted of presenting 61 different pictures, one at a time, at the centre of the screen, and the subject had to decide whether each picture had already been seen in the first part or not. Fifty percent of pictures were already presented, and 50% were new. The new pictures were taken from the pre-tested pool, and belonged to the same categories of the already presented pictures. Among the pictures already presented, 15 were the congruent images and 16 were the incongruent images, equally distributed to the four categories (i.e., unease, sublimity, vitality, and neutral). The experiment script is available on the OSF platform (https://osf.io/mxf8b/).

#### Affective state

To measure the current affective state a short form of the Positive and Negative Affect Scale (i.e., I-PANAS-SF) developed by Thompson^45^ was used in this study. It contains 10 items about different affective states (e.g., “Right now I feel attentive.”), and the subject has to answer to each of them on a five-point Likert scale from “not at all” to “extremely”. The score of the scale is computed separately for positive and negative affectivity.

#### Emotional intelligence

The Emotional Intelligence Scale^46^ was used to assess the participants' emotional intelligence. The scale includes 33 items that should be rated using a five-point Likert scale (1 = "I strongly disagree" to 5 = "I strongly agree"). An example item is as follows: “I know what other people are feeling just by looking at them”.

#### Demographic variables

Age, gender, education, and musical background (i.e., musical status, years of music experience) were assessed with a questionnaire.

#### Music liking and familiarity

We asked participants to rate on a scale from 1 to 4 how much they liked the musical excerpts presented, as previous studies suggested that this variable can influence emotional experience^47^. Furthermore, we assessed whether participants were familiar with the musical excerpts, as also familiarity seems to play a role in musical emotions experience^48^.

### Procedure

On the starting page of the Survey platform (LimeSurvey 2.64.1), participants were informed of the nature of the tasks. Then, demographic information was collected, followed by the emotional intelligence and the PANAS questionnaires. At the end of the questionnaires, participants were redirected to the platform Pavlovia, where the memory experiment began. The entire duration was around 25 min. Written informed consent was obtained from all the study participants at the beginning of the study.

The current study was approved by the ethics committee of University of Innsbruck (certificate of good standing n. 31/2021), and was conducted in accordance with the declaration of Helsinki.

### Analysis

Based on the Signal Detection Theory^49^, we computed *d*′ for accuracy. In order to do so, *hits*, *misses*, *correct rejections*, and *false alarms* were computed for every participant, and separately for congruent and incongruent pictures. Hits refer to correctly identified “old” pictures, whereas misses refer to the not recognizing “old” pictures. Correct rejections refer to correctly identifying “new” pictures, whereas false alarms refer to incorrectly judging a new picture as “old”. Note that, to compute two distinct *d*′ values for congruent and incongruent pictures, we had to use the same correct rejections and false alarms twice (gathered from the “new” pictures), as in our experiment the “old” (i.e., congruent and incongruent) and “new” pictures were presented together in a unique recognition phase. We then analysed whether accuracy differed between congruent and incongruent pictures via a dependent sample t-test on the *d*′*-*values.

Finally, to examine the role of predictor variables relating to individual differences, we computed correlations between recognition accuracy (*d*′) and the predictor variables, namely, affective state (i.e., PANAS scores), emotional intelligence, years of music training, gender, music familiarity and music liking. The dataset is available online in the OSF platform (https://osf.io/3mhqc/).

## Results

We first checked whether participants’ performance in the recognition task was above chance, via a one sample t-test on the *d*′. The performance (*M* = 1.41, *SD* = 0.54) was significantly above chance, *t*(199) = 36.80, *p* < 0.001.

**Figure 2:**
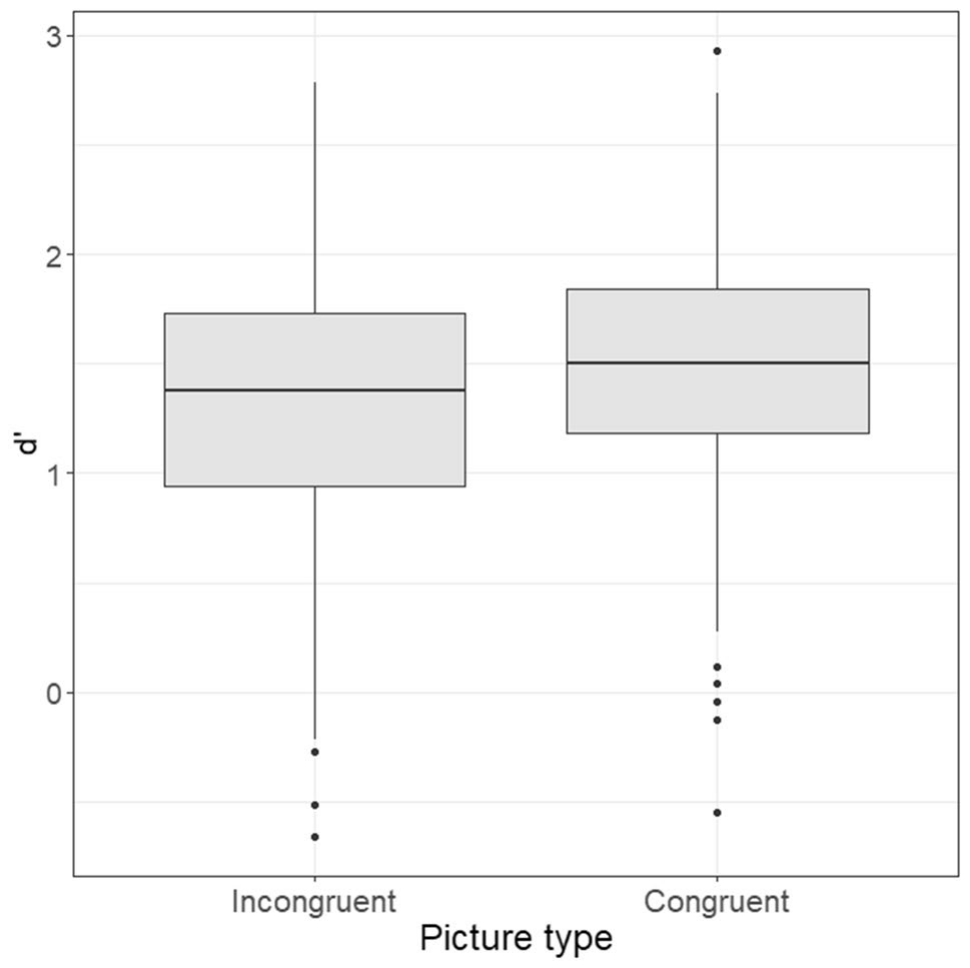
Boxplots representing recognition-accuracy (*d*′) for congruent and incongruent pictures. The horizontal line inside the box represents the median. The dots represent the outliers.

To answer the main hypothesis, we compared the accuracy (i.e., *d*′) for congruent pictures and incongruent pictures, through a dependent sample t-test. The t-test was significant, *t*(199) = 3.81, *p* < 0.001, *d* = 0.38, indicating that recognition-accuracy for congruent pictures (*M* = 1.48, *SD* = 0.56) was higher than that for incongruent pictures (*M* = 1.35, *SD* = 0.63), a result from now on referred to as “emotion-congruency effect” (instead of “mood-congruency effect”, as this would imply that the emotion is felt, something that our results alone study alone cannot guarantee). See Fig. 2.

To assess the possible impact of listener characteristics, we examined correlations between the emotion-congruency effect and the individual difference variables. No correlation emerged (see Table 1); therefore, we did not proceed any further in investigating whether these variables could predict the emotion-congruency effect (e.g., via a multiple linear regression).

**Table 1:** Correlations between the emotion-congruency effect and individual variables.

	1	2	3	4	5	6	7
1. Congruency effect	_						
2. PANAS pos	0.01	_					
3. PANAS neg	0.05	− 0.08	_				
4. EI	0.05	0.28**	− 0.17*	_			
5. Gender	0.11	− 0.04	− 0.004	0.05	_		
6. Music experience (yrs)	0.03	0.09	0.01	0.005	0.10	_	
7. Music liking	0.01	0.09	− 0.02	0.06	0.16*	0.20**	_
8. Music familiarity	0.04	0.01	0.02	− 0.04	− 0.02	0.16*	0.18*

Congruency effect is computed as the difference between the accuracy (*d*′) for congruent pictures and incongruent pictures.

*p > .05; **p > .01.

## Discussion

The present study aimed to assess whether music-evoked emotions could be indirectly assessed via a paradigm that leverage mood-congruency effects. This assumption is based on the fact that attention and memorization are enhanced when the affective state of an individual matches of emotional nature of a stimulus^9^. Thus, if the participants of the present study experienced emotions while listening to music, we expect them to have better recognition of emotionally congruent pictures than incongruent ones. Two-hundred participants completed an online task, where they listened to 15 music excerpts, each followed by emotional congruent pictures and incongruent pictures. After this part, participants performed a recognition task, with 50% of old pictures and 50% of new pictures. Results indicated that participants were more accurate in recognizing the emotionally congruent pictures than the emotionally incongruent ones (i.e., emotion-congruency effect). In what follows, we will first discuss whether using mood-congruency paradigms can be useful in the assessment of music-evoked emotions. Secondly, we will discuss the (lack of) effects of the individual variables assessed and mention the implications of our findings for the use of music in mood-congruent paradigms. Finally, we will describe the limitations and possible applications of the current study.

The present findings suggest that using a paradigm based on mood-congruency effects may provide a viable alternative or additional method for ascertaining the presence of specific music-evoked emotions. This suggestion is based on an interpretation of the observed emotion-congruency effect from the standpoint of the mood-congruency theory^21^, according to which listeners would have felt emotive states while listening to the music. There is ample evidence to suggest that music can be used to induce emotion and that emotion elicitation can occur within a few seconds only^22,23^. Of particular note is the finding that when listeners indicated to have felt emotions belonging to four different GEMS-categories (sublimity, vitality, tension, and sadness), the feelings tended to correlate with emotion-specific brain activation patterns^9^. In light of this evidence, it is not unreasonable to suggest that our results were driven by participants feeling the different emotions conveyed by the music (i.e., tension, sublimity, and vitality).

Concerning individual differences, the fact that the assessed individual variables were not related with the emotion-congruency effect suggests that current mood, gender, music experience, emotional intelligence, music liking and familiarity did not influence the superior memory for emotionally congruent pictures over incongruent ones. These results are interesting in light of previous studies on music-evoked emotions. While some findings showed that certain personality traits, such as empathy, musical expertise or mood lead to stronger and more differentiated emotions^32,33,39,50^, in some cases these differences did not emerge^51^. The reason for which musical experience and expertise were not related with the observed congruency effect is unclear, and could be due to a lack of variability in degree of musical expertise (our sample included only eight participants who considered themselves as professional musicians), or to the lack of a sufficiently nuanced assessment of musical expertise (such as through years of formal training). Concerning the other individual variables that we collected, it is possible that, either the variables, or the induced emotions, did not vary substantially across participants; in fact, the music excerpts we chose were already selected because of their ability to evoke certain emotions^9,41^. However, it is also possible that emotion-congruency effects are so robust as to override the effect of individual differences. In support of this reading, Mayer et al.^52^ found that the strength of the emotions felt after a mood induction had no influence on mood-congruency effects (i.e., both strong and weak emotions led to emotion-congruent content being perceived and remembered more readily).

Finally, our findings add to evidence suggesting that emotion-congruency effects do occur in response to music-evoked emotions. Music was often used to induce an emotional states^22^, but less often to test mood-congruency effects. If this effect reflects a genuine mood-switch in the participants, music could be thus used to assess mood-congruency effects, as an alternative to the more commonly used non-musical mood induction procedures. Previous studies often used different techniques, such as asking participants to write about a positive or negative emotional event that happened in their lives^10^, presenting emotional faces^53^, giving participants a bag of candies^14^, or performing hypnosis^54^. Alternatively, instead of inducing an emotional state, some studies simply recorded the actual mood of the participants at the moment of the experiment^13^. Using music would have the advantage of being an easy, enjoyable approach, and an objective stimulus (i.e., everybody listens to the same music).

### Limitations

 Even though previous evidence makes it seem likely^9,23^ that our participants felt the emotions conveyed by the music, the present study alone cannot conclusively prove that participants experienced a change in felt emotion rather than a change in perceived emotion. In fact, it is possible that what we observe is more generally an “emotion”-congruency effect rather than a mood-congruency effect, where the emotion is only recognized but not felt. In the latter event, the effect would be the mere result of a cognitive interpretation of the music’s emotional connotation, for example, with a music piece recognized as being “joyful” and thus priming the attention towards affectively related pictures. Musical stimuli can be indeed used as affective primes (implying emotion recognition rather than induction). However, previous studies using the affective priming paradigm, used mainly single musical elements (e.g., chords) that are very short (e.g., < 1 s)^55–57^. When using longer stimuli (e.g., melodies) in these paradigms, these effects could be the result of felt rather than recognized emotion. Support for this possibility has been offered by a recent study on affective priming, which used Western Classical music excerpts of 20 s of duration^58^. At any rate, the extent to which affective priming and congruency effects are a result of felt or recognized emotion is an important matter for future studies that could use brain-imaging techniques to ascertain the presence of specific emotional states.

Secondly, the present experiment was conducted online because of COVID-19 restrictions, therefore we had no control over the environment in which the participants found themselves, when taking the study. Nevertheless, the accuracy rates were above chance performance, suggesting that participants were actually engaged in the task. However, future studies might want to replicate the current results in a more controlled environment. In addition, in the present study we used only Western Classical music, and results cannot be generalized to other music genres. Finally, due to design limitations, we could not look at the contribution of type of emotion to accuracy outcomes, nor was it possible to compare the accuracy for neutral pictures with that for emotional pictures. Further studies could be specifically designed to examine accuracy separately for each emotion category (e.g., by providing more trials per category). This could reveal whether congruency effects are stronger with specific emotions (e.g., with positive emotions^59^).

To conclude, using a paradigm based on mood-congruency effects could be a way to assess behaviourally if music can evoke specific emotions. Moreover, researchers interested in studying mood-congruency effects could choose to use music to evoke different emotional states in the same group of people. Finally, these findings can also be relevant in the clinical field, such as with mood and anxiety disorders. For example, a music-based intervention could be applied to the cognitive bias modification paradigm, where the goal is to change the patient’s selective information processing, that is usually biased towards negative stimuli^60^. If “happy” music can induce happy feelings and influence consequent cognitive processes, it could become a helpful tool when treating these disorders. As a first step, future studies could uncover whether the present results are also replicable with mood/anxiety disorders’ patients.

## Supplementary Information

Supplementary Information.
