# Supplementary material for: Musical emotions affect memory for emotional pictures
Source: Sci Rep. 2022 Jun 23;12:10636. doi: 10.1038/s41598-022-15032-w (PMC9219376; doi:10.1038/s41598-022-15032-w)
Supplement: Supplementary file 1 — Supplementary Information. [file 41598_2022_15032_MOESM1_ESM.docx]

Supplementary information

Music excerpts used

Sublimity:

- Brahms, Johannes: Intermezzo for piano, Op. 117
- Tschaikovski, Peter: Rokoko-Variations, Variation No. 8
- Fauré, Gabriel: Ballade for piano and orchestra, Op. 19
- Mendelson, Bartholdy Felix: Song Without Words, op.19, No 1
- Chopin, Piano Concerto No. 1 in E minor, Op. 11 - II. Romance - Larghetto

Vitality:

- Delibes, Léo: Coppélia. Ballet in 3 Acts. 1st act, Prelude
- Milhaud, Darius. Scaramouche. Brazileira
- Strauss, Johann. Chit-Chat-Polka
- Bach, Johann Sebastian: Piano Concerto, No. 3, 1st movement.
- Dvořák: Symphony №9, "From The New World" - IV - Allegro Con Fuoco

Tension:

- Martinů, Bothuslav: String Quartet, No 3, H 183, Vivo
- Kurtag, György: String Quartet, Op. 1: II. Con Moto
- Chopin, Prelude in G minor Opus 28
- Luigi Boccherini, Fandango
- Bartok, Bela: Piano Sonata BB88, 1st movement
